# Combined targeting of MDM2 and CDK4 is synergistic in dedifferentiated liposarcomas

**DOI:** 10.1186/s13045-017-0482-3

**Published:** 2017-06-19

**Authors:** Audrey Laroche-Clary, Vanessa Chaire, Marie-Paule Algeo, Marie-Alix Derieppe, François L. Loarer, Antoine Italiano

**Affiliations:** 1 Université de Bordeaux, Bordeaux, France; 2 Institut National de la Santé et de la Recherche Medicale (INSERM) U1218, Institut Bergonié, 229 cours de l’Argonne, 33076 Bordeaux cedex, France; 3 Department of Medical Oncology, Institut Bergonié, Bordeaux, France

**Keywords:** MDM2, CDK4, Well-differentiated/dedifferentiated liposarcomas

## Abstract

**Purpose:**

*MDM2* and *CDK4* are frequently co-amplified in well-differentiated/dedifferentiated liposarcoma (WDLPS/DDLPS). We aimed to determine whether combined MDM2/CDK4 targeting is associated with higher antitumour activity than a single agent in preclinical models of DDLPS.

**Experimental design:**

DDLPS cells were exposed to RG7388 (MDM2 antagonist) and palbociclib (CDK4 inhibitor), and apoptosis and signalling/survival pathway perturbations were monitored by flow cytometry and Western blotting. Xenograft mouse models were used to assess tumour growth and survival. Treatment efficacy was assessed by Western blotting, histopathology and tumour volume.

**Results:**

RG7388 and palbociclib together exerted a greater antitumour effect than either drug alone, with significant differences in cell viability after a 72-h treatment with RG7388 and/or palbociclib. The combination treatment significantly increased apoptosis compared to the single agents. We then analysed the in vivo antitumour activity of RG7388 and palbociclib in a xenograft model of DDLPS. The combination regimen reduced the tumour growth rate compared with a single agent alone and significantly increased the median progression-free survival.

**Conclusions:**

Our results provide a strong rationale for evaluating the therapeutic potential of CDK4 inhibitors as potentiators of MDM2 antagonists in DDLPS and justify clinical trials in this setting.

## Introduction

Liposarcoma (LPS) is the most common soft tissue sarcoma (STS), accounting for up to 25% of all sarcomas in adults [8]. LPSs can be divided into three categories based on their cytogenetic characteristics: myxoid/round cell LPS, pleomorphic LPS and well-differentiated and dedifferentiated LPS (WDLPS/DDLPS). WDLPS represents more than 40% of all diagnosed cases of LPS and can be subdivided into three main histological subtypes: adipocytic, sclerosing and inflammatory. The three main locations of WDLPS are the limbs (50%), retroperitoneum (30%) and paratesticular area (20%) [10]. Although these tumours do not metastasize, retroperitoneal and paratesticular tumours are associated with a high risk of local recurrence (up to 90%) and dedifferentiation (up to 20%) [4, 19], whereas these risks are lower for tumours located in the limbs [17].

Dedifferentiated liposarcoma (DDLPS) represents the most frequent sarcoma subtype. Despite an optimal locoregional treatment, the local recurrence rate can reach 80%, and distant metastatic relapse occurs in up to 30% of cases [19, 24].

Systemic therapy is the most suitable approach for patients with advanced/unresectable disease. However, we have reported that the role of conventional chemotherapy in this setting is very limited, with an objective response rate of only 12% and a median PFS of 4.6 months [14]. New therapeutic options are therefore urgently needed.

We have shown that DDLPS cells contain supernumerary ring or giant marker chromosomes composed of highly amplified sequences from the 12q14-15 chromosomal region [8, 13]), which contain the MDM2 (12q15) and HMGA2 (12q14.3) genes. We have also shown that the CDK4 gene (12q14.1) belongs to a distinct amplicon, which is inconsistent but present in up to 90% of cases [13].

Recently, a class of imidazoline compounds was identified as potent and selective inhibitors of the TP53-MDM2 interaction [28]. These molecules, termed nutlins, interact specifically with the TP53-binding pocket of MDM2 and thus release TP53 from negative control. Treating cancer cells that express wild-type TP53 with nutlins stabilizes TP53 and activates the TP53 pathway, leading to the activation of TP53 target genes, cell cycle arrest, apoptosis and/or senescence. We have recently demonstrated that nutlins that are specific inhibitors of the TP53-MDM2 interaction activate the TP53 pathway and decrease cell proliferation in patients with WDLPS/DDLPS [25]. However, only a few patients exhibited an objective response or long-term stable disease [6, 25].

Considering the important function of CDK4 in the cell cycle, CDK4 gene amplification and overexpression very likely play important roles in DDLPS tumourigenesis. Oral highly selective CDK inhibitors, including palbociclib, ribociclib, and abemaciclib, represent an important therapeutic advancement in oncology particularly in the field of advanced breast cancer [30]. The CDK4-targeting agent palbociclib is well tolerated but achieves only limited activity as a single agent, with only 1 patient out of 60 exhibiting an objective response in a recent phase 2 study of patients with advanced DDLPS [6].

Here, we report the first study aimed at determining whether targeting both the MDM2-TP53 interaction and CDK4 has a synergistic effect on DDLPS.

## Materials and methods

### Cell lines

**Fig. 1:**
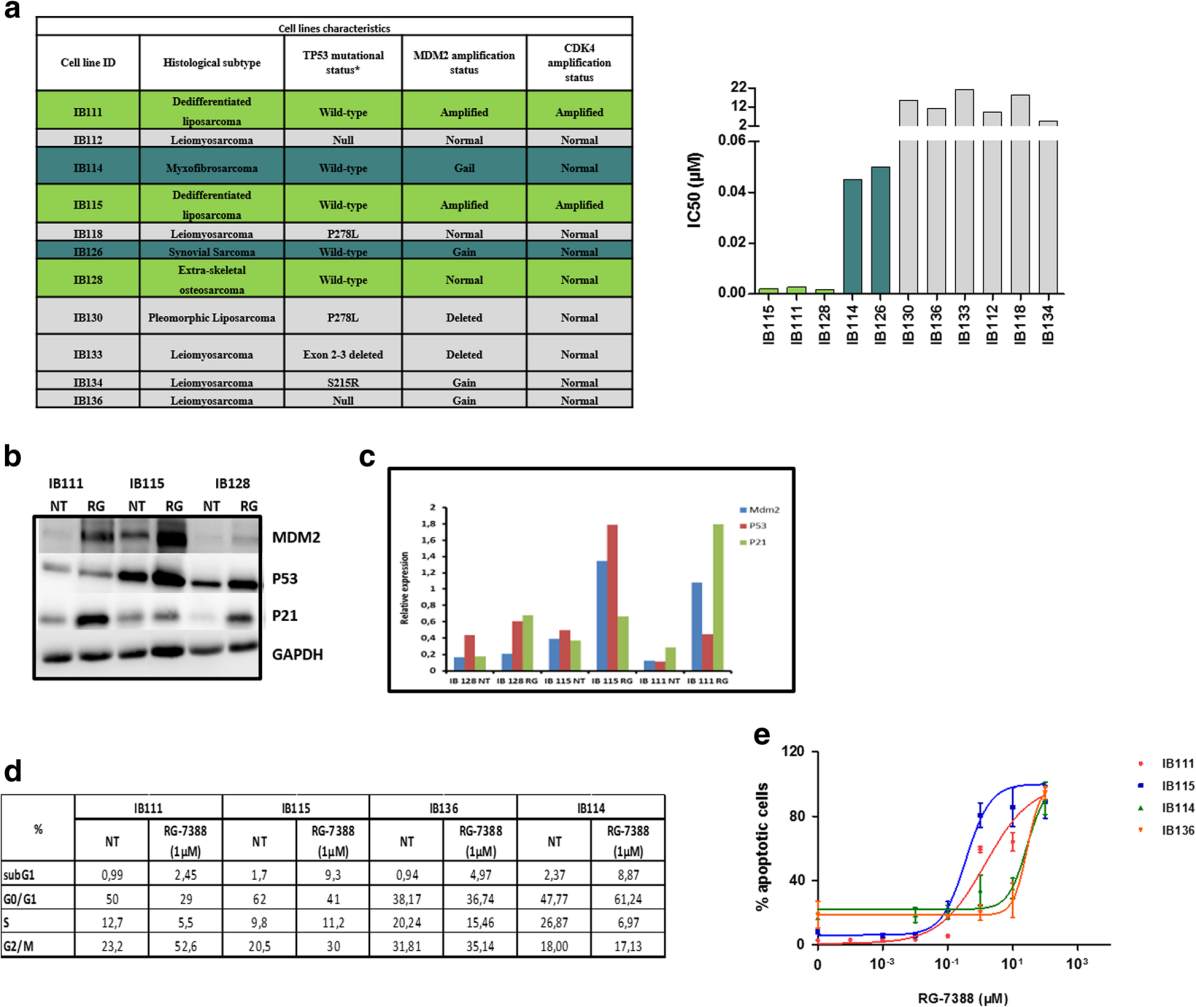
**a** Characteristics of the 11 cell lines and IC50 (μM) of RG-7388 in these cells. IB111, IB115, IB128, IB114 and IB126 express wild-type TP53, and the other cell lines contain TP53 mutations. MDM2 is only amplified in IB115 and IB111 cells. **b** Sensitive cells were untreated or exposed to the IC50 of RG-7388 (*RG*) and then immunoblotted for MDM2, TP53 and P21. The immunoblots are quantified in **c. d** Cell cycle profile before and after treatment with 1 μM nutlin, as analysed by PI incorporation and flow cytometry after 48 h of treatment. **e** Effect of nutlin on apoptosis in four cell lines: two very sensitive (IB111 and IB115), one sensitive (IB114) and one resistant cell line (IB136)

The cell lines used in this study were derived from human surgical STS specimens in the laboratory of Pr Jean-Michel Coindre and Dr Frédéric Chibon (Institut Bergonié, Bordeaux, France) after obtaining patient consent (Fig. 1a) [16]. All cell lines were cultured in RPMI 1640 medium containing GlutaMAX™ supplement (Life Technologies), 10% (*v*/*v*) foetal bovine serum (FBS), 1% penicillin/streptomycin and 0.2% Normocin (InvivoGen) at 37 °C with 5% CO_2_. The cells were routinely passaged every 2 or 3 days.

### Study reagents

RG7388 (MDM2-TP53 interaction inhibitor) was supplied by Roche, and palbociclib (CDK4 inhibitor) was purchased from Selleck Chemicals (Houston, TX, USA). For cell treatments, the culture medium was replaced, and the cells were incubated with different concentrations of drugs for 72 h, as indicated in figure legends.

### Cell viability assay

Cells were seeded in triplicate wells in 96-well plates at a density of 5000 cells/well, cultured in fresh growth medium for at least 24 h and treated with increasing concentrations of drugs for 72 h. After the drug-treatment period, 2-deoxyglucose (2-DG) or 3-(4,5-dimethylthiazol-2-yl)-2,5-diphenyltetrazolium bromide (MTT, Sigma-Aldrich Chimie, Saint-Quentin-Fallavier, France) was immediately added to the wells at a final concentration of 0.5 mg/mL, and the cells were incubated for 3 h. Then, the supernatant was discarded, 100 μL of dimethyl sulfoxide (DMSO, Sigma-Aldrich Chimie, Saint-Quentin-Fallavier, France) was added and the absorbance was monitored using a Flexstation 3 Plate reader (Bio-Tek Instruments, Colmar, France) at 570 nm with 630 nm as reference. The IC_50_ was calculated with GraphPad Prism software version 5.00 for Windows (GraphPad Software, La Jolla, CA, USA). Each experiment was repeated at least three times.

### Cell apoptosis assay

Cells (2 × 10^5^/well) were seeded in six-well plates and treated for 72 h with several specific drug concentrations. After treatment, the cells were washed once with PBS and labelled with annexin V-FITC and propidium iodide (PI) according to the manufacturer’s protocol (BD Biosciences, Erembodegem, Belgium). Then, the cells were analysed with a FACSCalibur flow cytometer (BD Biosciences, Erembodegem, Belgium). The percentages of cells in early apoptosis (annexin V positive, PI negative) and in late apoptosis or necrosis (annexin V and PI positive) were calculated using FlowJo version 7.6.3 for Windows (Tree Star Inc, Ashland, OR, USA). The data are presented as the mean ± SEM of three independent experiments.

### Western blotting

Treated and control cells were harvested in 60 μL of radio-immunoprecipitation assay (RIPA) lysis buffer [9]. The lysate was centrifuged (13,000 rpm, 15 min, 4 °C), and the supernatant was stored at −80 °C. Equal amounts of total protein (30 μg) were electrophoresed on 8, 12 or 15% sodium dodecyl sulfate (SDS) polyacrylamide gels and transferred onto polyvinylidene difluoride (PVDF) membranes. The blots were probed overnight at 4 °C with an anti-phospho Rb (ser 807/811, 1/1000, OZYME), an anti-Rb (1/500, OZYME), anti-TP53 (1/200, Santa Cruz Biotechnology), anti-MDM2 (1/50, Calbiochem), anti-P21 (1/33, Calbiochem) or anti-GAPDH (1/5000, Santa Cruz Biotechnology) primary antibody diluted in PBST (100 mM phosphate, 27 mM KCl, 1.37 M NaCl, pH 7.4 after 1× dilution; 0.2% Tween-20) with 5% BSA. The horseradish peroxidase-conjugated secondary antibody (Santa Cruz Biotechnology) was diluted 1:5000. Bound antibodies were visualized by Fusion FX7 (Fisher Bioblock Scientific, Waltham, MA, USA) using Imobilon™ Western (Millipore Corporation, Billerica, MA, USA), an enhanced chemiluminescence detection kit. The resulting bands were analysed and quantified using ImageJ® 1.49g software (National Institutes of Health, Bethesda, MD, USA). GAPDH served as a loading control. Each membrane was reused two times after desaturation in glycine buffer (6.6 M, pH 2) at 56 °C for 30 min. Each experiment was repeated at least twice.

### Proteomic analysis

Cells were trypsinized, washed in cold PBS and lysed by 3 cycles of freezing in liquid azote and thawing on ice as described by Tansey [26]. The cellular extracts were then centrifuged at 13,000 rpm at 4 °C for 10 min, and the supernatant was incubated with benzonase. We performed three separate protein extractions. The proteins in each cell line (IB115 and IB111) were analysed by label-free quantitative proteomic analysis as previously described [11].

### Drug synergy assays

Cells were treated with a single drug or a combination of two drugs for 72 h. To confirm synergistic effects between the two drugs, a diagonal constant ratio combination design was developed according to that proposed by Chou and Talalay [3]. The cells were incubated with twofold serial dilutions at several concentrations above and below the IC_50_ of the two drugs at a constant ratio. After the incubation period, MTT was immediately added to the wells, and the absorbance was monitored using a Flexstation 3 Plate reader. The synergy analysis was performed using the isobologram and combination index (CI) methods derived from the median-effect principle of Chou and Talalay. The combination effects of the two agents can be summarized as follows: CI <1 (under the curve), synergistic; CI = 1 (near the curve), additive; and CI >1 (above the curve), antagonistic. The synergy experiments were repeated at least three times.

### Animal studies

Four- to 5-week-old female Ragγ2C−/− mice were used. Xenograft tumours were induced by subcutaneous injection of 0.2 mL of a cell suspension containing 5 × 10^6^ live IB115 cells into the right flank of each mouse. This study followed the Spanish and European Union guidelines for animal experimentation (RD 1201/05, RD 53/2013 and 86/609/CEE). The mice were randomized into control and treatment groups (*n* = 8 for vehicle and treatment groups) 2 weeks after the tumours became measurable (15 days after injection; day 1 of treatment). The mice were randomized into four groups: vehicle, nutlin alone (100 mg/kg, oral gavage five times per week), palbociclib alone (130 mg/kg, oral gavage five times per week), and both drugs (nutlin and palbociclib five times per week at 100 and 130 mg/kg, respectively). Palbociclib was administered in sodium lactate (50 mM, pH 4), and RG7388 was administered in a vehicle solution supplied by Roche. The tumours were measured every 2–3 days with callipers, and the diameters were recorded. Tumour volume was calculated using the following formula: *a* ^2^ *b*/2, where *a* and *b* are the two largest diameters. The mice were sacrificed by cervical dislocation 1 week after treatment ended, and the tumours were collected for histopathological analyses. Progression-free survival curves were established based on a twofold increase in tumour volume as the event. All experimental manipulations with mice were performed under sterile conditions in a laminar flow hood.

### Statistical analysis

The data were analysed using Student’s *t* test for comparisons of two means and ANOVA followed by Tukey’s multiple comparison test for comparisons among more than two groups; all experiments were repeated in duplicate or triplicate. The data are presented as the mean ± SD, and significant differences are indicated as *p < 0.05, **p < 0.01 and ***p < 0.001.

Progression-free survival was analysed using a log-rank test (Mantel-Cox test).

## Results

### RG7388 activates the TP53 pathway, significantly inhibits proliferation and induces cell cycle arrest and apoptosis in DDLPS cell lines

As predicted by the mechanistic model of TP53 regulation, RG7388 significantly inhibited the proliferation of five STS cell lines without TP53 mutations, as assessed by Sanger sequencing (IC50 2–50 nM), but not of six other STS cell lines that contained TP53 mutations (Fig. 1a). The most sensitive cell lines were the DDLPS cell lines IB111 and IB115, characterized by MDM2 gene amplification, and the extraskeletal osteosarcoma cell line IB128, with no alteration in MDM2 copy number. In agreement with the mechanism of RG7388 action, RG7388-treated DDLPS cell lines showed an accumulation of TP53 protein and its targets, P21 and MDM2, as revealed by Western blotting (Fig. 1b, c).

One of the main cellular functions of activated TP53 is the blocking cell cycle progression in the G1 and G2 phases. Treatment of exponentially proliferating IB111 and IB115 DDLPS cell lines with RG7388 for 48 h led to a dose-dependent cell cycle block in G2/M phase (Fig. 1d). Another primary function of activated TP53 is to induce apoptosis. Exposure of exponentially proliferating IB111 and IB115 DDLPS cell lines to RG7388 RO5503781 for 72 h led to the induction of apoptosis in a dose-dependent manner, as revealed by an annexin V assay (Fig. 1e). These cell cycle and apoptosis effects were not observed in the myxofibrosarcoma IB114 and leiomyosarcoma IB136 cell lines used as controls.

### Palbociclib, a selective CDK4/6 inhibitor, inhibits Rb phosphorylation and blocks proliferation in DDLPS cells

**Fig. 2:**
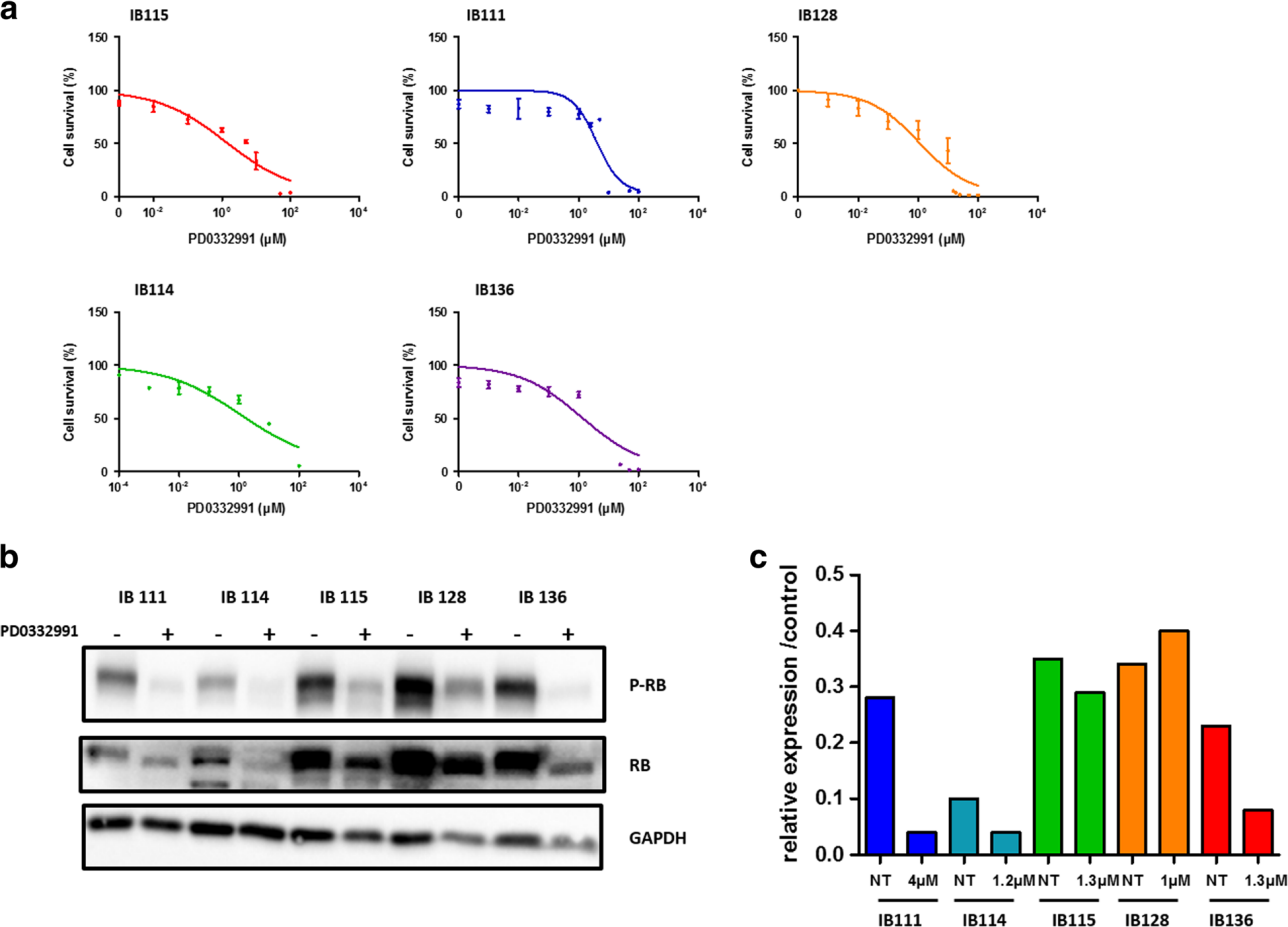
**a** Effect of PD0332991 on dedifferentiated liposarcoma (IB115 and IB111), myxofibrosarcoma (IB114), leiomyosarcoma (IB136) and osteosarcomas (IB128) cell lines. The IC50 values are 1.3, 4, 1.2,1.3 and 1 μM, respectively. **b** Western blot analysis of p-Rb, Rb total and GAPDH in the 5 cell lines, which were either untreated or exposed to PD0332991. **c** Quantification of Western blot analyses for IB111, IB114, IB115, IB128 and IB136 cells treated or not at the IC50 of PD0332991

**Fig. 3:**
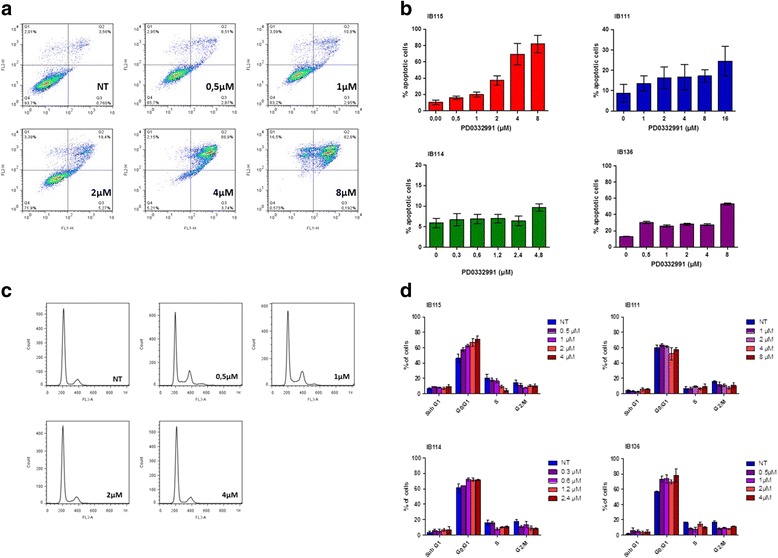
**a** Effect of PD0332991 on IB115 cell death using annexin V-FITC and propidium iodide staining followed by flow cytometry. **b** Quantification of apoptosis in the four cell lines, which were untreated or exposed to PD0332991. **c** Cell cycle profile of the B115 cell line before and after treatment with 0.5, 1, 2 or 4 μM PD0332991, as analysed by PI incorporation and flow cytometry. **d** Quantification of cell cycle analysis for IB115, IB111, IB114 and IB136 cells

Palbociclib is a novel, selective CDK4/6 inhibitor in clinical development. To demonstrate its impact on DDLPS cells in vitro, we examined the effects of palbociclib in our IB111 and IB115 DDLPS cell lines, as well as in the control cell lines IB114 and IB136 and in IB128 cells, an osteosarcoma cell line. Palbociclib reduced Rb phosphorylation with an IC50 between 1 and 4 μM in IB111, IB115, IB136 and IB114 cells but not in IB128 cells (Fig. 2a, b). Correspondingly, palbociclib decreased IB111 and IB115 DDLPS cell growth in a dose-dependent manner, and IB111 cells were less sensitive than IB115 cells (IC50, 4 for IB111 and 1.3 for IB115) (Fig. 2c). An annexin V assay demonstrated that exposing exponentially proliferating DDLPS cell lines to palbociclib for 72 h also led to the induction of apoptosis in a dose-dependent manner (Fig. 3a, b), and this effect was more pronounced in IB115 cells than in IB111 cells. A cell cycle analysis in IB115 cells demonstrated cell cycle arrest at G0–G1 phase and a decreased proportion of cells in S phase following 48 h of exposure to increasing doses of palbociclib (Fig. 3c). No induction of apoptosis was observed in the myxofibrosarcoma IB114 cell line used as a control, whereas palbociclib induced apoptosis in the leiomyosarcoma IB136 cell line.

**Fig. 4:**
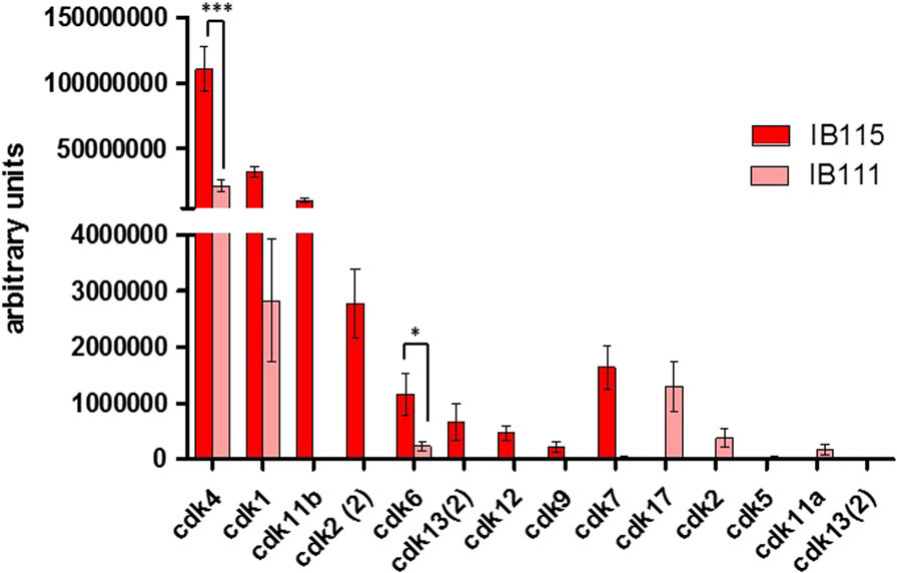
CDK protein expression was measured by the quantitative proteomic label-free LCMS/MS method; the analysis was performed on three independent samples for each cell line

The sensitivity differences between the two dedifferentiated LPS cell lines could be explained by the basal expression of CDKs, particularly the expression of CDK4 and CDK6, which was significantly lower in IB111 cells than in IB115 cells (Fig. 4).

### CDK inhibitors sensitize DDLPS cells to nutlin-induced apoptosis

**Fig. 5:**
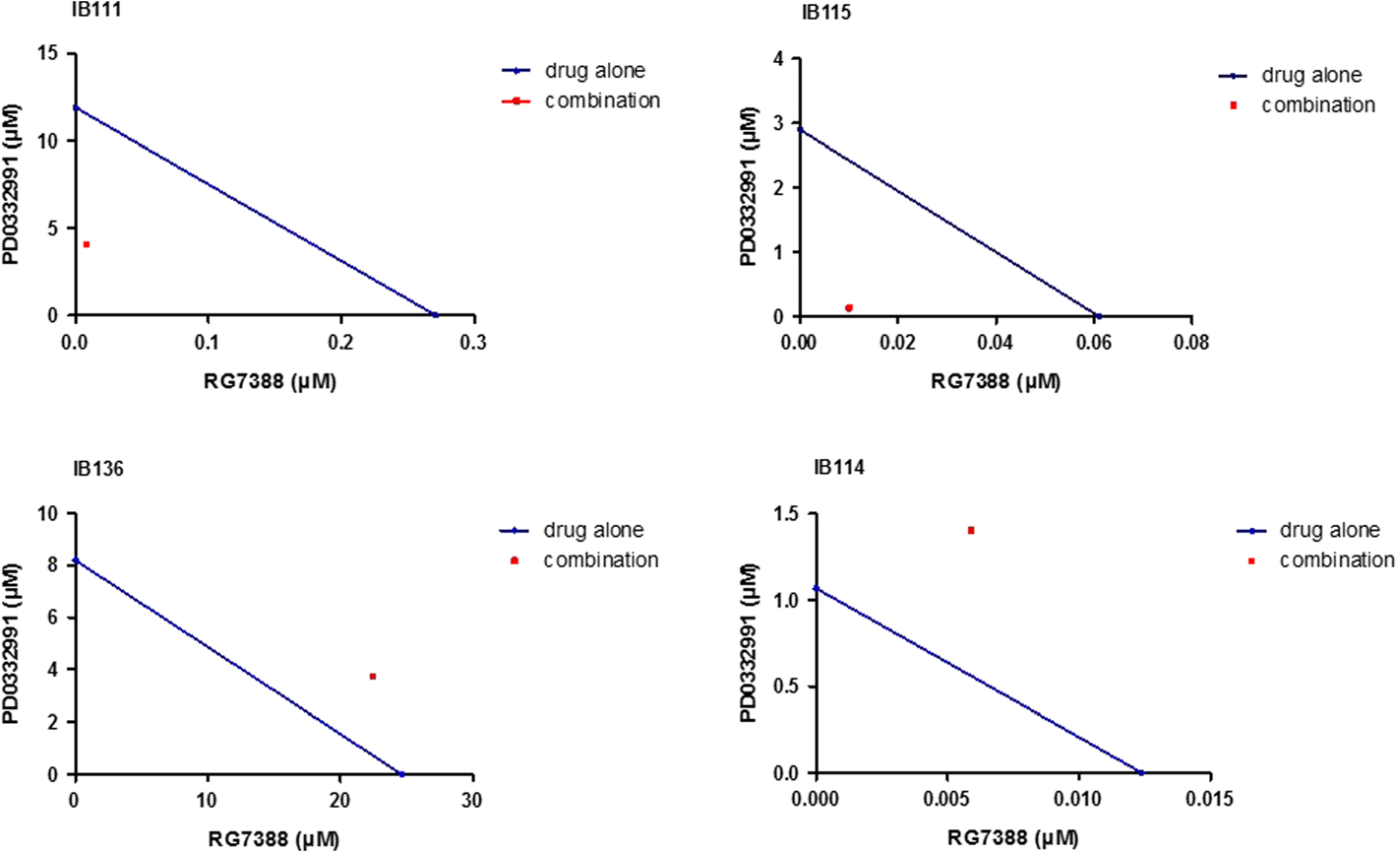
Isobologram representation for the IB111, IB115, IB136 and IB114 cell lines. The *dots* located at the *lower left*, on the *diagonal line*, or at the *upper right* indicate synergism, additivity and antagonism, respectively. The combination index (*CI*) was calculated to be 0.37, 0.2, 1.36 and 1.78, respectively

Since CDK inhibitors have been shown to sensitize tumour cells to RG7388-induced apoptosis, we investigated whether combined treatment with palbociclib and RG7388 results in greater antitumour activity than that observed with either drug alone. IB115, IB111, IB114 and IB136 cells were exposed for 72 h to different combinations of both agents at a constant ratio of 1; RG7388 and palbociclib were mixed and diluted serially (using twofold serial dilutions at several concentrations above and below the IC50 for the two drugs). The CIs were determined according to Chou and Talalay [3]. As shown in Fig. 5, we observed strong synergism in DDLPS cells but not in other histotype cells. Indeed, combining an MDM2 antagonist and a CDK4 inhibitor resulted in an antagonistic effect in myxofibrosarcoma (IB114) and leiomyosarcoma (IB136) cells, indicating that the synergistic effect was specific to the DDLPS histological subtype (Fig. 5).

**Fig. 6:**
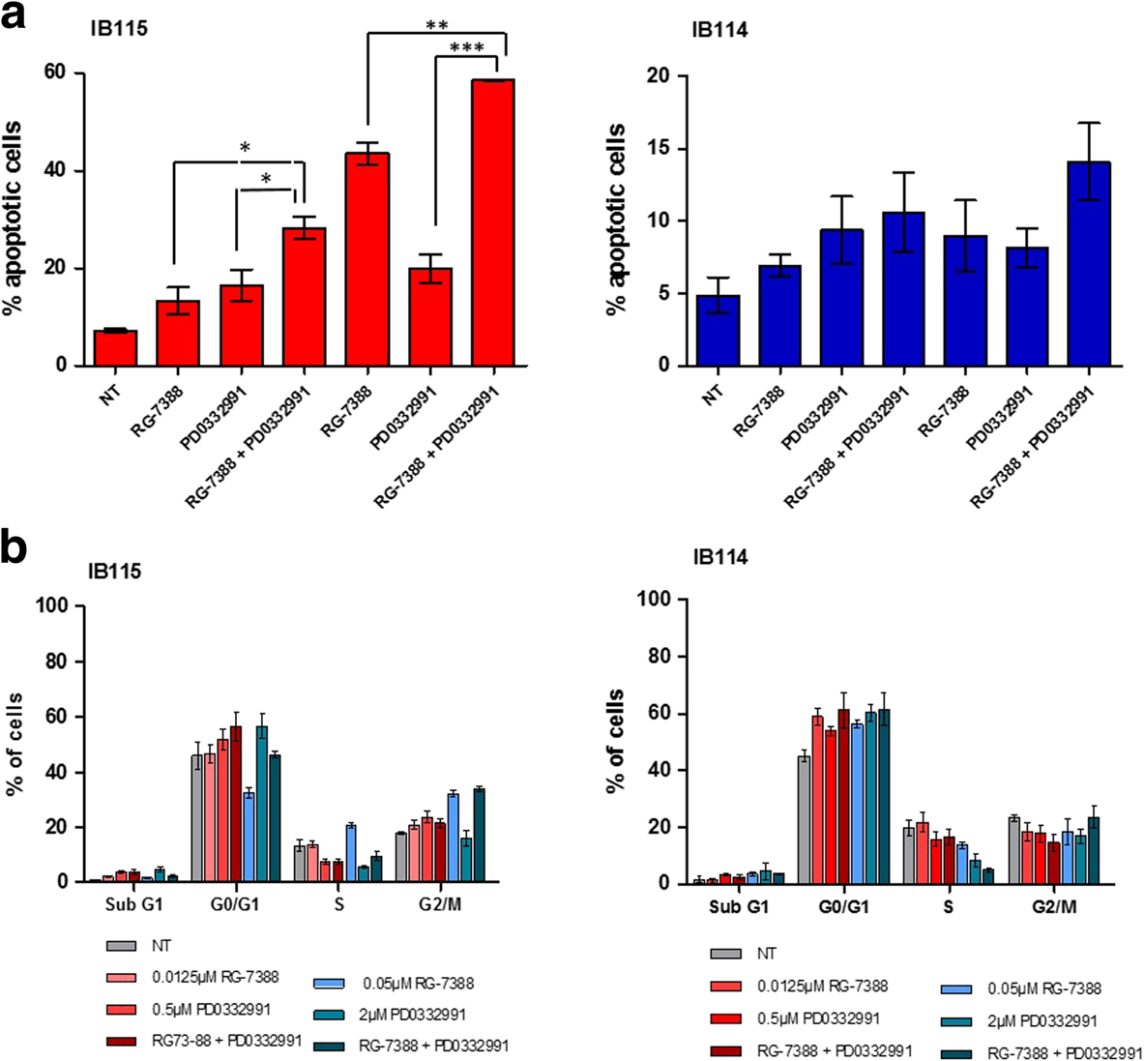
Treatment of IB115 (DDLPS) and IB114 (MFH) cells with nutlin and/or a cdk4 inhibitor induces apoptosis. **a** Cells were incubated with RG-7388 and/or PD0332991, and the annexin V-positive fractions were measured by flow cytometry at 72 h. The results are expressed as the mean ± SEM. **b** The effects of the single drugs alone and the two-drug combination on the cell cycle were measured by flow cytometry

Consistent with these observations, a quantitative apoptosis assay using flow cytometry revealed that a significantly increased percentage of DDLPS cells responded to treatment with a combination of RG7388 and palbociclib. Seventy-two hours after treatment, the DDLPS cells treated with the drug combination became mostly annexin V positive (from 43% apoptotic cells to 60%) compared with those treated with RG7388 or palbociclib alone (from 20 to 60%; see Fig. 6a). However, no effect was observed in other histotype cells (Fig. 6b).

**Fig. 7:**
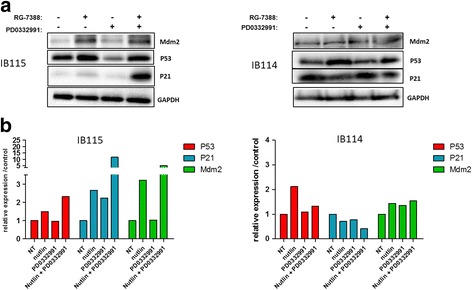
**a** Western blot analysis of the TP53 protein pathway in IB115 and IB114 cells, which were either untreated or exposed to 2 μM PD0332991 and/or 0.05 μM nutlin. **b** Quantification of Western blot analyses; the experiments were performed in duplicate

To confirm that the synergism of the RG7388 and palbociclib combination is TP53 dependent, we examined the synergistic induction of TP53 signalling. In DDLPS cells, we observed a significant increase in the protein levels of key TP53-regulated genes, such as P21 and MDM2, when the two drugs were combined versus treatment with a single agent alone (Fig. 7a). This effect was not observed in other histotype cells (Fig. 7a, b).

### In vivo activity of RG7388 and palbociclib against tumour growth

**Fig. 8:**
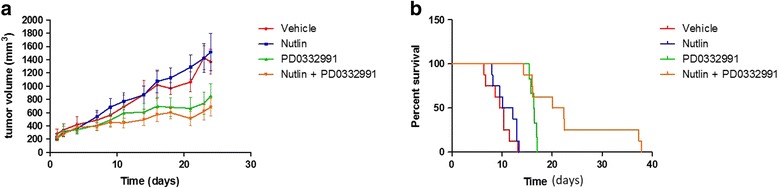
In vivo evaluation of the effect of combining nutlin and PD0332991. **a** Tumour growth in mice injected with IB115 cells and treated with either vehicle, nutlin, PD0332991 or a combination of the two drugs. There were eight mice in each group. **b** Event-free analysis: for each mouse, the tumour volume was monitored, and a twofold increase in tumour volume was considered to be an event

To further validate the in vitro study, we performed an in vivo study to determine the antitumour effects of the RG7388 and palbociclib combination. Xenograft tumours were generated by subcutaneous injection of IB115 cells in Ragγ2C−/− mice. The mice were randomized into four groups and treated for 3 weeks. These groups included control, RG7388 (RG7388 alone, 100 mg/kg by oral gavage five times a week), palbociclib (palbociclib alone; 130 mg/kg by oral gavage five times a week) and a combination of both drugs. After 3 weeks of treatment, we observed a significant effect on progression-free survival (evaluated as the time span from the beginning of treatment to the doubling of the initial tumour volume). The median time to doubling was 21.2 days for the combination treatment group, 11.1 days for the RG7388 group (*p* < 0.0001) and 16.3 days for the palbociclib group (*p* = 0.04) (Fig. 8b). After 3 weeks of treatment, the mice were sacrificed, and the tumours were extracted, weighed and evaluated by histopathology. No signs of toxicity were observed with the combination treatment.

## Discussion

WDLPS/DDLPS is the most frequent subtype of STS, and these tumours are characterized by a specific amplification of MDM2 (12q14-15) [8, 23]. Stimulating the tumour suppressor activity of wild-type TP53 has long been shown to eradicate tumour cells in animal models, which makes TP53 an attractive target for drug development. Inhibition of the MDM2­TP53 interaction with synthetic molecules might therefore lead to the accumulation of active TP53, followed by the apoptosis of tumour cells, as shown in vitro [2]. Nutlins represent a class of antagonists that inhibit the MDM2-TP53 complex, and they were first described in 2004. This class of agents can disrupt the MDM2-TP53 interaction by binding to a well-defined hydrophobic cleft of MDM2, the TP53 binding site, thereby liberating functional TP53 [2]. Limited preclinical data are available regarding the antitumour activity of nutlins in sarcomas [18, 21, 29]. Most of these studies were performed in bone sarcoma cell lines and not in STS models. We have recently shown that an MDM2 antagonist activates the TP53 pathway and decreases cell proliferation in patients with MDM2-amplified LPS. However, in this study, 25% of the patients displayed primary resistance [22].

The combination of genotoxic drugs with non-genotoxic nutlins has been reported to synergistically activate TP53 functions, paving the way for the potential use of nutlins in combinatorial drug therapy [5, 15]. Ohnstad et al. [20] investigated the in vitro effect of nutlin in combination with current cytotoxic drugs (e.g. doxorubicin, cisplatin and methotrexate) on sarcoma cells [20]. Among the drugs tested, only doxorubicin is used in the management of WDLPS/DDLPS. The authors found a synergy that supported the development of this combination in the clinics. We have recently reported that in a phase 1 study, combination therapy with doxorubicin and the nutlin compound RG7112 resulted in an apparent potentiation of TP53 activation in an unselected population of patients with advanced STS [1]. However, due to the toxicity profile of doxorubicin, this combination resulted in a high rate of grade 3 or 4 haematological toxicity, with 60% of patients experiencing severe neutropenia and 45% experiencing thrombocytopenia, precluding its future development.

Therefore, combining nutlin with a targeted non-genotoxic drug may represent a more relevant approach. Deregulation of the cyclin D-CDK4/6-Rb axis is a characteristic feature of WDLPS/DDLPS. Indeed, we previously reported that WDLPS/DDLPS is characterized by frequent amplification of the CDK4 gene, which occurs in 90% of cases, and consistent overexpression of CCND1 [12]. Therefore, inhibitors of this pathway represent a promising class of effective treatments for WDLPS/DDLPS. Palbociclib (PD0332991) is the first highly selective CDK4/6 inhibitor to be evaluated in humans and has been approved for the treatment of breast cancer [27]. We demonstrate here that the combination of palbociclib and an MDM2 antagonist, RG7388, activates the antiproliferative and proapoptotic functions of TP53 synergistically. Our results indicate that although RG7388 activates TP53, this agent alone is not sufficient to fully activate the TP53 response. The use of palbociclib significantly enhances the RG7388-dependent induction of TP53 activity. Nevertheless, the effect of the combination is strictly dependent on nutlin-3a; palbociclib only weakly activates TP53, and an antagonistic effect, rather than synergy, was observed in cell lines that are only slightly sensitive (IB114) or resistant (IB136) to RG7388. Further studies are required to elucidate the mechanisms involved in the synergy between nutlin and palbociclib. However, previous studies have suggested that Rb status is a critical factor influencing the cellular response to nutlin, with hypophosphorylated Rb favouring apoptosis and/or cell cycle arrest of nutlin-treated tumour cells [7].

In conclusion, our data provide a strong rationale for evaluating the therapeutic potential of CDK4 inhibitors as potentiators of MDM2 antagonists in WDLPS/DDLPS. A phase 1 trial assessing the safety and efficacy of the MDM2 antagonist HDM201 combined with the CDK4/6 inhibitor LEE001 has recently started and is currently ongoing in patients with advanced WDLPD/DDLPS. Given the devastating nature of this disease, the confirmation of our data in the clinical setting would represent a strong achievement for the sarcoma community.

## Abbreviations

GAPDH: Glyceraldehyde 3 phosphate dehydrogenase
LPS: Liposarcoma
STS: Soft-tissue sarcoma
WDLPS/DDLPS: Well-differentiated/dedifferentiated liposarcomas
